# Weight loss before total joint arthroplasty using a remote dietitian and mobile app: study protocol for a multicenter randomized, controlled trial

**DOI:** 10.1186/s13018-020-02059-w

**Published:** 2020-11-13

**Authors:** Michael W. Seward, Brielle J. Antonelli, Nancy Giunta, Richard Iorio, Wolfgang Fitz, Jeffrey K. Lange, Vivek M. Shah, Antonia F. Chen

**Affiliations:** Department of Orthopaedic Surgery, Brigham and Women’s Hospital, Harvard Medical School, Boston, USA

**Keywords:** Obesity, Obesity, Morbid, Weight loss, Knee osteoarthritis, Osteoarthritis, Hip, Mobile applications, Smartphone, Telemedicine, Nutritionists, Arthroplasty, Dietitian, Preoperative Period, Randomized Controlled Trial, Multicenter Study

## Abstract

**Background:**

The months prior to elective surgery may present an opportunity for patients to initiate behavior changes that will simultaneously ready them for surgery and improve their overall health status. An upcoming elective total joint arthroplasty (TJA) may serve as motivation for patients with severe obesity (body mass index [BMI]> 40 kg/m^2^) to lose weight, as it may optimize clinical outcomes following TJA and help them become eligible for TJA since some surgeons use a BMI of 40 kg/m^2^ as a cut-off for offering surgery in an effort to optimize outcomes.

**Methods:**

The purpose of this multicenter randomized, controlled trial is to assess the feasibility and efficacy of a 12-week remote dietitian (RD) supervised dietary and physical activity weight loss intervention and mobile app for 60 patients with severe obesity prior to undergoing TJA. Intervention participants will receive access to a smartphone app and connect with an RD who will contact these participants weekly or bi-weekly via video calls for up to nine video calls. Together, participants and RDs will set goals for lifestyle modifications, and RDs will check on progress towards achieving these goals using in-app tools such as food logs and text messages between video calls. All patients will be encouraged to lose at least 20 pounds with a goal BMI < 40 kg/m^2^ after 12 weeks. Individuals randomized to the control group will receive clinical standard of care, such as nutritionist and/or physical therapy referrals. Outcome and demographic data will be collected from blood serology, chart review, mobile app user data, pre- and postintervention surveys, and phone interviews. The primary outcome measure will be weight change from baseline. Secondary outcome measures will include percentage of patients eligible to undergo TJA, number of sessions completed with dietitians, self-reported global health status (PROMIS Global Health scale), self-reported joint-specific pain and function (Knee injury and Osteoarthritis Outcome Score (KOOS) or Hip disability and Osteoarthritis Outcome Score (HOOS)), and serologies such as hemoglobin A1c, total lymphocyte count, albumin, and transferrin. Qualitative responses transcribed from phone interviews about the intervention will also be analyzed.

**Discussion:**

This will be the first study to assess pre-operative weight loss in patients with severe obesity anticipating orthopaedic surgery using an RD and mobile app intervention aimed at helping patients become eligible for TJA.

**Trial registration:**

Registered on 1 April 2020 at Clincialtrials.gov. Trial number is NCT04330391.

## Background

Surgery is a major decision for many patients and often involves reflection on personal health behaviors when weighing the risks and benefits of procedures [1, 2]. Positive preoperative lifestyle factors including nonsmoking status, increased physical activity, and normal body mass index (BMI) are associated with improved postoperative outcomes [3–5]. However, time pressure, fiscal constraints, and limited provider experience encouraging lifestyle changes may hamper effective counseling during the preoperative period [6, 7]. Digital technologies have recently been proposed as a potential solution to the barriers of preoperative lifestyle counseling [2].

A previous remote dietitian (RD) intervention for overweight pregnant women in 2018 demonstrated high satisfaction with dietitians and a desire for an integrated mobile app before a motivating event like childbirth [8]. An upcoming elective surgery may be similarly motivating for patients to make lifestyle modifications, especially for patients with severe obesity who may become eligible for surgery if they reduce their BMI below the 40 kg/m^2^ maximum for total joint arthroplasty (TJA) used by some surgeons as a cutoff for offering surgery in an effort to optimize outcomes [9].

A recent retrospective review of patients with severe obesity (BMI > 40 kg/m^2^) who lost weight before total knee arthroplasty (TKA) found that losing at least 20 pounds before surgery was associated with fewer discharges to a facility and shorter lengths of stay, but losing just five or 10 pounds had no effect [10]. A 2018 study of physical activity after TKA found a combined dietitian and financial incentive intervention increased step counts and physical activity following TKA [11].

However, despite multiple investigations regarding the association between lifestyle interventions and postoperative outcome, there is a dearth of research regarding success of weight loss interventions specifically prior to TJA. Although there are three studies of pre-operative dietary interventions prior to TJA, only one study concluded before surgery and this study did not compare pre-operative weight loss to a control group [12–14]. A 2015 review of nonsurgical, non-pharmacologic weight loss interventions in patients with obesity prior to TJA found that there was limited research on the association between weight loss prior to TJA and pre- and post-operative outcomes, and that future studies should address the safety, optimal timing, duration, amount of targeted weight loss, and delivery of pre-TJA interventions [15]. In addition, a recent study of patients about to undergo TKA or who were recently post-TKA found that patients wanted a weight loss program that started before surgery, was at least 6 months in duration, and focused on both diet and exercise [16].

In our proposed mixed-methods, multicenter randomized controlled trial (RCT), our primary aim is to assess the feasibility and efficacy of a 12-week RD supervised dietary and physical activity weight loss intervention and mobile app for patients with severe obesity prior to undergoing TJA. We hypothesize that a 12-week RD supervised dietary and physical activity weight loss intervention and mobile app for patients with severe obesity who are TJA candidates will result in (1) greater weight loss than clinical standard of care, (2) a higher percentage of patients eligible to undergo TJA by having a BMI below the standard cutoff of 40 kg/m^2^ within 3 months of enrollment, and (3) a higher percentage of patients undergoing TJA within 6 months of enrollment.

## Methods/design

### Study design

**Fig. 1:**
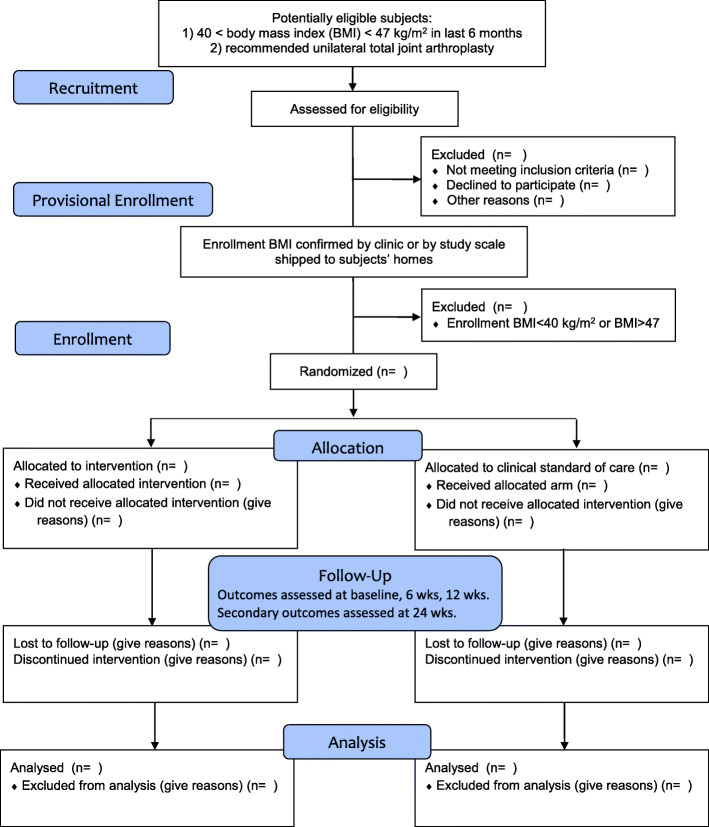
Flow diagram. Randomized controlled trial design

This mixed-methods study is a prospective, two-arm, randomized controlled trial that will take place remotely and involve patients at two institutions. The trial includes a 12-week intervention period followed by a 3-month follow-up period for selected secondary outcomes. Various outcomes will be assessed at baseline, 6 weeks, 12 weeks, and 24 weeks (Fig. 1).

### Eligibility criteria

#### Inclusion criteria

1. Subjects must be greater or equal to 18 years of age.
2. Subjects must have 40 kg/m^2^ < BMI < 47 kg/m^2^ recorded in the electronic medical record within 6 months prior to enrollment. Weights recorded earlier than 2 weeks before enrollment must be verified by an additional weight measurement using study scales sent to subjects’ homes or in clinic during enrollment.
3. Subjects must have end-stage knee or hip osteoarthritis, avascular necrosis, or rheumatoid arthritis with recommended primary, unilateral TJA by orthopaedic surgeons at two institutions.
4. Subjects would consider undergoing TJA if eligible.
5. Subjects own a smartphone, tablet, or personal computer compatible with video calls and a specified mobile app. The device must have a camera, access to Internet, and Apple App Store or Google Play.
6. All subjects must be willing to comply with the requirements of the study and provide informed consent prior to enrollment. Evidence of a signed and dated informed consent document indicating that the subject (or a legally acceptable representative) has been informed of all pertinent aspects of the study must be obtained prior to data collection.

#### Exclusion criteria

1. Subject’s enrollment BMI is less than 40 kg/m^2^ or greater than 47 kg/m^2^ when verified by study scale at subjects’ homes or by weight recorded at enrollment in clinic. BMI over 47 kg/m^2^ would require inappropriate weight loss of over three pounds per week to reach a BMI of 40 kg/m^2^ after 12 weeks.
2. Subjects unable to comprehend and speak English.
3. Subjects undergoing revision TJA.
4. Subjects undergoing bilateral TJA.
5. Subjects planning to undergo bariatric weight loss surgery in the next 6 months.
6. Subjects planning to be pregnant in the next 6 months.
7. Subjects unwilling or unable to use a smartphone, tablet, or personal computer with home Internet access.
8. Subjects are incarcerated.
9. Subjects are pregnant women or vulnerable individuals.

### Patient population and recruitment procedure

Patients of fellowship trained arthroplasty surgeons at two institutions will be identified as potentially eligible for the study based on 40 kg/m^2^ < BMI < 47 kg/m^2^ recorded in the patient’s medical record within the last 6 months and recommended primary, unilateral total knee or hip arthroplasty. For patients identified as potentially eligible while in clinic, an orthopaedic surgeon will introduce the patient to the study, and a member of the research team (research assistant or trainee) will conduct an eligibility screening and obtain verbal consent either in-person or by phone. For patients identified as potentially eligible outside of clinic, a member of the research team will call the patient to introduce the study, and conduct eligibility screening and consent on the phone.

Eligible, interested, and consented patients will be provisionally enrolled, and all subjects will be shipped a standard bathroom scale to their home (Fig. 1). This initial enrollment is provisional, as subjects will be required to verify enrollment BMI by (a) submitting a photo to the study team of their study scale during a weigh-in that displays their body weight, or by (b) recording a baseline weight at an onsite healthcare facility within 2 weeks of provisional enrollment. Once enrollment BMI is verified to be 40 to 47 kg/m^2^, subjects will be fully enrolled and randomized to the intervention arm or clinical standard of care arm via REDCap. Subjects will be given a $40 check for enrolling, which includes (1) taking a baseline survey, (2) recording the baseline weight at an onsite healthcare facility or with a home study scale, and (3) getting baseline labs within 2 weeks of the baseline weight. Baseline weights will be recorded without shoes or outer clothing (e.g., coats). All subjects will be given a $20 check if they can verify a mid-study weight during week 6 of the study by recording a weight at any onsite healthcare facility or by submitting home study scale photos. All subjects will receive a $40 check for completing an end-of-study survey, weight, and labs at 10–14 weeks after enrollment.

### Ethics

The trial protocol has been approved by the primary institution’s Human Research Committee (protocol number: 2020P000322). The protocol has been registered on ClinicalTrials.gov (ID: NCT04330391). All participants will provide verbal and/or written consent prior to enrollment in the study.

### Randomization and allocation

Enrolled subjects will be randomized into two groups with an allocation ratio of 1:1 in blocks of four. The allocation table will be randomly generated via Microsoft Excel by an independent third-party clinical researcher, uploaded to the study REDCap randomization model, and will not be accessible to study staff within REDCap. Group assignment will be revealed via the REDCap model only at the time of randomization during enrollment following informed consent procedures and enrollment BMI verification.

### Arms

Intervention participants will download a telemedicine online/smartphone app (Nutrimedy, Brookline, MA, USA) and be connected at enrollment with a certified registered dietitian who will contact intervention participants weekly or bi-weekly via video calls and unlimited in-app text messaging for up to 3 months. The first week will include either one 55-min video session or two 25-min sessions. Weeks 2–4 will have weekly 25-min video calls, and weeks 5–12 will have biweekly 25-min sessions for a total of 8–9 sessions over 12 weeks. Together, participants and dietitians will come up with goals for the 12 weeks, and dietitians will check on progress toward these goals using in-app tools such as food logs and messaging between video calls. All patients will be encouraged to lose at least 20 pounds with a goal BMI < 40 kg/m^2^ after 12 weeks.

Dietitians will use principles of motivational interviewing, cognitive behavioral interventions, and the socioecological model to remotely provide behavior and lifestyle interventions. Motivational interviewing is a patient-centered, collaborative approach to counseling in which the dietitian elicits personally relevant reasons from subjects to make behavior changes that may promote weight loss. Dietitians and patients then structures goals for lifestyle modifications around these personal motivations [17]. Cognitive behavioral interventions provide subjects with strategies to change cognitive processes to alter behaviors, such as self-monitoring (e.g., recording food intake, weight, and exercise), stimulus control (e.g., keeping less healthy foods in cabinets out of sight), and accountability by having frequent check-ins with dietitians [18]. The socioecological model proposes that multiple levels of influence including intrapersonal (e.g., knowledge and attitudes), interpersonal (e.g., family and friends), and community factors (e.g., organizations, workplaces, neighborhoods) can impact energy-balance behaviors and weight outcomes [19]. Dietitians will promote the adoption and maintenance of behaviors across multiple levels of influence.

Individuals randomized to the clinical standard of care group will receive care according to current clinical guidelines. This may include a physical therapist and/or nutritionist referral, of which several programs within the institutions are offered to patients interested in weight loss, including the Nutrition Wellness Service (NWS) and Program for Weight Management (PWM).

### Blinding

Any staff involved in weighing participants or obtaining labs, as well as the principal investigator will be blinded from the group assignment until statistical analyses are finished. The subjects will not be blinded due to the nature of the study. While adverse events (AEs) are unexpected given the minimal risk of the study, if an AE potentially related to the intervention occurs, the principal investigator will decide whether the group assignment for a particular subject should be revealed.

### Surveys

All participants will complete a baseline survey at enrollment, and an end-of-study survey 10–14 weeks after enrollment (Additional files 1, 2 and 3). Questions about weight loss methods and the level of interest and confidence in weight loss were taken from Cleveland et al. [20]. Questions about motivation for weight loss were adapted from Meyer et al. [21]. Questions about exercise, diet, and goal setting routines were taken from the Paving Wheel Wellness score by Frates [22]. Items querying food frequency, hours spent doing physical activity, demographics, and opinions about the intervention were adopted from Seward et al. [8]. Alcohol and tobacco use questions were taken from the AUDIT-C alcohol screen and World Health Organization Global Tobacco Surveillance System [23, 24]. Surveys also included the Lower Extremity Activity Scale (LEAS) [25], PROMIS-Global Health scale, and the Knee injury and Osteoarthritis Outcome Score (KOOS) or Hip disability and Osteoarthritis Outcome Score (HOOS) depending on the joint [26, 27]. In addition to the demographic information collected on baseline surveys, we will also review each subject’s electronic medical record to obtain medication lists.

### Outcome measurements

Outcome and demographic data will be collected from blood serology, chart review, mobile app user data, pre- and postintervention surveys, and phone interviews.

#### Primary outcome measurement

The primary outcome will be weight change at 12 weeks (10–14 weeks) after study enrollment. Weights will be recorded at an onsite healthcare clinic or by home study scales.

#### Secondary outcome measurement

##### Weight and related measures

Weight-related secondary outcomes will include: percent weight (kg) change, BMI change, percentage of patients losing at least 20 pounds, percentage of patients eligible to undergo TJA by BMI < 40 kg/m^2^ at 3 months after study enrollment, number of weeks (up to 6 months) after enrollment until eligible for TJA by BMI < 40 kg/m^2^, number of dietitian calls until eligible for TJA by BMI < 40 kg/m^2^, and weight at 3-month post-operative visit when available. The percentage of patients undergoing TJA within 6 months of study enrollment will be calculated by review of each subject’s electronic medical record.

##### Feasibility and interactivity

The number of completed video call sessions with dietitians, number of food log uploads, and number of in-app messages sent to dietitians will be recorded by the mobile app.

##### Behaviors and function

Several behavioral and functional measures described previously will be assessed via surveys (Additional files 1, 2 and 3). When available, we will also review each subject’s electronic medical record to obtain standard clinical assessments including the PROMIS-Global Health scale, pain and function using Knee injury and Osteoarthritis Outcome Score (KOOS) or Hip disability and Osteoarthritis Outcome Score (HOOS).

##### Labs

Blood serologies measuring hemoglobin A_1c_, fasting blood sugar, fructosamine, insulin; serum albumin, pre-albumin, transferrin, total lymphocyte count; creatinine, and blood urea nitrogen (BUN); iron, folate, vitamin B12, serum electrolytes, and vitamin D will be recorded at baseline and end-of-study. Hemoglobin A_1c_, total lymphocyte count, albumin, and transferrin are highly recommended to evaluate nutrition status before orthopaedic procedures [28].

##### Qualitative

Intervention subjects at enrollment will be asked if they would be interested in completing a brief 5-min interview in-person or by phone at the end of the study (10–14 weeks after enrollment) to discuss qualitative feedback about the intervention. At the end of the intervention, interested intervention participants will be interviewed using an interview script (Table 1). Responses will be transcribed verbatim during the interview for later analysis.

**Table 1:** Interview guide to be used for follow-up phone interviews with intervention subjects

	Domain	Question
1	Goals	What helped you achieve your goals during this study?
2	Motivation	What motivated you to achieve your goals?
3	Health Coach	How would you describe your experience with the mobile app and the dietitian?
4	Communication	What did you think about the different components for delivery of the intervention including video calls, phone calls, food logs, and the mobile app.
5	Communication	What did you think about the frequency of contact with dieticians?
6	Improvements	How would you improve the intervention?
7	Other	Any last comments about the intervention?

### Sample size calculation

The 2016 paper by Gandler et al. found a pre-operative dietitian intervention before TJA resulted in a weight loss of − 3.38 kg (standard deviation [SD] 6.62) versus a weight gain of + 2.01 kg (SD 6.45) in the usual care group [13]. Using 80% power to detect a difference between weight loss of − 3.38 kg in the intervention group versus + 2.01 kg weight gain in the usual care group with SD 6.62 kg, this study would require 24 patients per arm. Thus, we will aim for 30 participants in each arm to allow for 25% dropout.

### Statistical analyses

The main statistical analysis will be performed via an intention-to-treat analysis. Per protocol sensitivity analysis (completing at least half or four video calls) will also be performed. The significance level for statistical tests will be set at *p* ≤ 0.05. Baseline characteristics between the two groups will be assessed using t-tests for continuous variables and chi-square or two-tailed Fisher’s exact tests (when required by small cell counts) for categorical variables. Differences over time in continuous variables between the intervention and clinical standard of care group, such as percent weight change, body weight, and BMI, will be analyzed using *t* tests. Missing week 12 weight outcomes will be carried forward from week 6 outcomes when available.

We will measure feasibility via the ease of recruitment based on the ratio of enrolled subjects to the number of people approached about the study, and via maintenance of contact with a dietitian (e.g., number of video calls completed) based on study participant attrition and follow-up survey completion rates (retention). We will measure acceptability via survey items about components of the intervention, and through qualitative comments from a semi-structured interview. We will perform survey analyses using SAS (SAS Institute Inc., Cary, NC) or similar software. For most questions, participants will choose between four categories: strongly agree, somewhat agree, somewhat disagree, and strongly disagree. Assuming the distribution of responses to baseline surveys will be relatively similar, we will pool follow-up survey answers into “agree” and “disagree” for simplicity, and then conduct chi-square or two-tailed Fisher’s exact tests to examine differences between intervention and control group responses to follow-up surveys.

Qualitative data will be analyzed using the immersion crystallization method [29]. This qualitative technique includes multiple rounds of “immersion” through close readings of transcripts, followed by reflection and the “crystallization” of emerging themes. Multiple investigators will read the transcripts and arrive at a unanimous consensus concerning the emerging themes. Then, one investigator will code the transcripts to organize representative quotes.

### Quality control and safety monitoring

Prior to the clinical trial, research staff will receive protocol training to confirm that staff understand the roles and process of the trial. One RD will deliver the intervention for all intervention subjects. The RD will be certified in clinical nutrition and lifestyle counseling (credentialed Registered Dietitian [RD] by the Academy of Nutrition and Dietetics, master’s degree [MS] in clinical nutrition, Licensed Dietitian/Nutritionist [LDN], and Certified Nutrition Support Clinician [CNSC]), and will have significant experience with the mobile app used for the intervention. The RD and mobile app staff are required to complete courses in Health Insurance Portability and Accountability Act (HIPAA) awareness for healthcare providers.

The study team will contact the RD every 3 weeks to review any safety concerns. We define unsafe weight loss as greater than 20 pounds of weight loss per month. All intervention participants will be asked to submit self-reported weights weekly through the mobile app, email, or REDCap survey. The RD will be instructed to promptly alert the study team of any participants meeting this criterion so that they may be removed from the study with appropriate referrals to their primary care physician who can guide treatment after seeing them in clinic or otherwise escalate their care according to their needs (e.g., admitting them to a hospital for a comprehensive workup). AEs will be tracked and reported per institutional review board (IRB) protocol.

Adherence to the IRB-approved protocol will also be reviewed at the participant safety meetings that occur every 3 weeks with the principal investigator. The principal investigator will have ultimate responsibility for the integrity, completeness, and confidentiality of all data. Surveys will be administered and stored using REDCap, a secure HIPAA compliant and password protected web-based application, and only study personnel will have access to the research participant data. Personnel from the study team will be designated for study management, which will include periodic monitoring of the study to ensure that proper records are kept, study procedures are followed, and complete and accurate data are collected.

## Discussion

This will be the first non-surgical trial to assess pre-operative weight loss in patients with severe obesity anticipating orthopaedic surgery to our knowledge, and the first study to evaluate a pre-operative weight loss intervention aimed at helping these patients become eligible for TJA. This will also be the first study to evaluate the utility of a RD and mobile app intervention before TJA.

There are three studies of pre-operative dietary interventions involving some amount of time prior to TJA, though just one measured pre-operative weight loss while the remaining two studies only measured post-operative weight as endpoints. Liljensøe et al. randomized 77 participants to a standard care control group or a dietician supervised weight loss group that consisted of nutrition education group sessions and a low calorie (810 kcal/day) diet using formula foods for 8 weeks before surgery, followed by a 1-year maintenance period after surgery [12]. Intervention participants lost an average of 10.7 kg in the 8 weeks before surgery, and the authors concluded that a pre-operative weight loss program shortly before TJA was safe to implement. The study did not measure pre-operative weight loss in the control group. Another study by Gandler et al. randomized 40 patients to either a weight loss intervention consisting of a minimum of four sessions with a dietician or usual care [13]. The intervention group lost 3.4 kg and had improved physical health scores compared to a 2.0 kg weight gain in the control group. However, it is unclear how many sessions occurred, and how many sessions occurred pre- versus post-operatively over the one-year study period; the author did not respond to requests for clarification. A third study by Pellegrini et al. randomized 16 participants to a 14-week weight loss program of up to 14 sessions that started on average 20 days before surgery (range 7–39 days) vs. 12 weeks postoperatively [14]. However, a maximum of only two pre-operative sessions were allowed for the pre-operative group, such that the pre-operative intervention was almost entirely post-operative. Both groups lost a similar amount of weight (2.5 kg) at 12 weeks after surgery, although most participants preferred to start the intervention prior to surgery.

This study has the potential to deliver a higher quality pre-operative weight loss intervention for patients than previously studied interventions, as it provides accessible and convenient dietary advice that is offered on a one-on-one basis, at more frequent time intervals, on-demand, and commencing earlier prior to surgery compared to methods described in previous studies. This study offers individualized one-on-one sessions as opposed to the group sessions offered in the Liljensøe et al. study, provides an average of three sessions with a dietitian every 4 weeks compared to at least four sessions over 1 year in the Gandler et al. study, and begins 12 weeks before surgery compared to an average of 20 days before surgery in the Pellegrini et al. study [12–14].

Considering the COVID-19 physical distancing recommendations and clinic suspensions, this study can provide important lessons on remote weight monitoring and nutrition services. While we intend to collect baseline and end-of-study laboratory data on subjects, collecting labs at both timepoints may not ultimately be feasible due to COVID-19. Remote weight monitoring will be the primary challenge conducting this study and will require sending study scales to subjects’ homes to verify recorded weights. Future studies may benefit from exploring open source smart scales that can more easily integrate with third-party mobile apps, allowing for frequent, verified weight measurements without sacrificing convenience for study participants.

## Supplementary Information

**Additional file 1.**

**Additional file 2.**

**Additional file 3.**

## Data Availability

The datasets used and/or analyzed during the future study will be available from the corresponding author on reasonable request.

## Abbreviations

BMI: Body mass index
EMR: Electronic medical record
HIPAA: Health Insurance Portability and Accountability Act
RCT: randomized, controlled trial
RD: Registered dietitian
THA: total hip arthroplasty
TJA: Total joint arthroplasty
TKA: Total knee arthroplasty

## References

[1] Warner DO (2009). Surgery as a teachable moment: lost opportunities to improve public health. Arch Surg..

[2] Robinson A, Slight R, Husband A, Slight S. The value of teachable moments in surgical patient care and the supportive role of digital technologies. Perioper Med (Lond). 2020;9:2. 10.1186/s13741-019-0133-z. 10.1186/s13741-019-0133-z PMC6998815 32042404

[3] Myers JN, Fonda H (2016). The Impact of Fitness on Surgical Outcomes: The Case for Prehabilitation. Curr Sports Med Rep..

[4] Khullar D, Maa J (2012). The impact of smoking on surgical outcomes. J Am Coll Surg..

[5] Choban PS, Flancbaum L (1997). The impact of obesity on surgical outcomes: a review. J Am Coll Surg..

[6] Grocott MPW, Plumb JOM, Edwards M, Fecher-Jones I, Levett DZH. Re-designing the pathway to surgery: better care and added value. Perioper Med (Lond). 2017;6:9. 10.1186/s13741-017-0065-4. 10.1186/s13741-017-0065-4 PMC5477682 28649376

[7] Williams K, Beeken RJ, Fisher A, Wardle J (2015). Health professionals’ provision of lifestyle advice in the oncology context in the United Kingdom. Eur J Cancer Care (Engl)..

[8] Seward MW, Simon D, Richardson M, Oken E, Gillman MW, Hivert M-F (2018). Supporting healthful lifestyles during pregnancy: a health coach intervention pilot study. BMC Pregnancy Childbirth..

[9] McElroy MJ, Pivec R, Issa K, Harwin SF, Mont MA (2013). The effects of obesity and morbid obesity on outcomes in TKA. J Knee Surg..

[10] Keeney BJ, Austin DC, Jevsevar DS (2019). Preoperative weight loss for morbidly obese patients undergoing total knee arthroplasty. J Bone Jt Surg..

[11] Losina E, Smith KC, Paltiel AD, Collins JE, Suter LG, Hunter DJ (2019). Cost-effectiveness of diet and exercise for overweight and obese patients with knee osteoarthritis. Arthritis Care Res (Hoboken).

[12] Liljensøe A, Laursen J, Bliddal H, et al. FRI0620-HPR Weight Loss Intervention Before Total Knee Arthroplasty – Feasibility and Safety. Ann Rheum Dis. 2015;74:1326.

[13] Gandler N, Simmance N, Keenan J, Choong PFM, Dowsey MM (2016). A pilot study investigating dietetic weight loss interventions and 12 month functional outcomes of patients undergoing total joint replacement. Obes Res Clin Pract..

[14] Pellegrini CA, Chang RW, Dunlop DD, Conroy DE, Lee J, Van Horn L (2018). Comparison of a patient-centered weight loss program starting before versus after knee replacement: a pilot study. Obes Res Clin Pract..

[15] Lui M, Jones CA, Westby MD. Effect of non-surgical, non-pharmacological weight loss interventions in patients who are obese prior to hip and knee arthroplasty surgery: a rapid review. Syst Rev. 2015;4:121. 10.1186/s13643-015-0107-2. 10.1186/s13643-015-0107-2 PMC4584125 26410227

[16] Pellegrini CA, Ledford G, Hoffman SA, Chang RW, Cameron KA (2017). Preferences and motivation for weight loss among knee replacement patients: Implications for a patient-centered weight loss intervention. BMC Musculoskelet Disord..

[17] DiLillo V, West DS (2011). Motivational interviewing for weight loss. Psychiatr Clin North Am..

[18] Kelley CP, Sbrocco G, Sbrocco T (2016). Behavioral modification for the management of obesity. Prim Care - Clin Off Pract..

[19] Raynor HA, Champagne CM (2016). Position of the Academy of Nutrition and Dietetics: Interventions for the Treatment of Overweight and Obesity in Adults. J Acad Nutr Diet..

[20] Cleveland LP, Seward MW, Simon D, Rifas-Shiman SL, Lewis KH, Bennett-Rizzo C, Halperin F, McManus KD, Block JP. BWHealthy Weight Pilot Study: A randomized controlled trial to improve weight-loss maintenance using deposit contracts in the workplace. Prev Med Rep. 2020;17:101061. 10.1016/j.pmedr.2020.101061. 10.1016/j.pmedr.2020.101061 PMC7011078 32071848

[21] Meyer AH, Weissen-Schelling S, Munsch S, Margraf J (2010). Initial development and reliability of a motivation for weight loss scale. Obes Facts..

[22] Frates B (2017). Measuring your Overall Wellness Using the PAVING Wheel. Paving the path to wellness. Harvard Health Publications.

[23] Bush K, Kivlahan DR, McDonell MB, Fihn SD, Bradley KA (1998). The AUDIT alcohol consumption questions (AUDIT-C): An effective brief screening test for problem drinking. Arch Intern Med..

[24] Global Adult Tobacco Survey Collaborative Group. Tobacco questions for surveys: a subset of key questions from the global adult tobacco survey (GATS), 2nd Edition [Internet]. Atlanta, GA; 2011. Available from: https://www.who.int/tobacco/surveillance/en_tfi_tqs.pdf. Accessed 10 Dec 2019.

[25] Saleh KJ, Mulhall KJ, Bershadsky B, Ghomrawi HM, White LE, Buyea CM (2005). Development and validation of a lower-extremity activity scale: Use for patients treated with revision total knee arthroplasty. J Bone Jt Surg - Ser A..

[26] Roos EM, Toksvig-Larsen S. Knee injury and Osteoarthritis Outcome Score (KOOS) - validation and comparison to the WOMAC in total knee replacement. Health Qual Life Outcomes. 2003;1(17). 10.1186/1477-7525-1-17 PMC161802 12801417

[27] Nilsdotter AK, Lohmander LS, Klässbo M, Roos EM. Hip disability and osteoarthritis outcome score (HOOS) - Validity and responsiveness in total hip replacement. BMC Musculoskelet Disord. 2003;4(10). 10.1186/1471-2474-4-10 PMC161815 12777182

[28] Cross MB, Yi PH, Thomas CF, Garcia J, Della Valle CJ (2014). Evaluation of malnutrition in orthopaedic surgery. J Am Acad Orthop Surg..

[29] Borkan J, Crabtree BF, Miller WL (1999). Crystallization-Immersion. Doing Qualitative Research [Internet].

