# Supplementary material for: Weight loss before total joint arthroplasty using a remote dietitian and mobile app: study protocol for a multicenter randomized, controlled trial
Source: J Orthop Surg Res. 2020 Nov 13;15:531. doi: 10.1186/s13018-020-02059-w (PMC7662734; doi:10.1186/s13018-020-02059-w)
Supplement: Supplementary file 1 — Additional file 1. [file 13018_2020_2059_MOESM1_ESM.pdf]

Implementing Weight Loss Before Total Joint Arthroplasty Using A Remote Dietitian and Mobile App: A Randomized, Control Trial

PRE-INTERVENTION QUESTIONNAIRE

· This survey asks about your opinions and experiences regarding lifestyle and weight loss. Your responses may help us to improve our intervention for current and future studies at Brigham and Women's Hospital.

· It will take approximately 15 minutes to complete this questionnaire.

· Read each question and answer it as best as you can. Remember, there are no right or wrong answers.

· Your answers will be kept completely confidential. We use a study identification number instead of your name on all our forms.

What is your Study ID number? ________________________________

During the last 3 months, did you actively try to lose weight?

- ◯ Yes
- ◯ No

How did you try to lose weight? (check all that apply.)

- ☐ Weighing yourself frequently
- ☐ Eating fewer calories
- ☐ Eating less fat
- ☐ Eating less carbs
- ☐ Exercising
- ☐ Eating breakfast daily
- ☐ Working to reduce stress
- ☐ Using meal replacements (liquid shakes or bar from companies like Slim Fast, Optifast, or HMR)
- ☐ Maintaining a consistent eating pattern throughout the week (eating similar food on weekdays and weekends)
- ☐ Using diet pills, laxatives, diuretics, water pills
- ☐ Purging or making yourself vomit
- ☐ Other (please specify)

Other method: ________________________________

---

1 Are you currently following a specific diet?

- ◯ Yes
- ◯ No

Which diet? (Please check all that apply.)

- ☐ Vegetarian or Vegan
- ☐ Atkins Diet
- ☐ South Beach Diet
- ☐ Ornish Diet
- ☐ Paleo Diet
- ☐ Mediterranean Diet
- ☐ Other low carbohydrate diet
- ☐ Other low fat diet
- ☐ My Fitness Pal, Lose it, or another web/mobile app
- ☐ Other

Please specify other diet: __________________________________

3 Have you seen a dietician or nutritionist in the last three months?
- ◯ Yes
- ◯ No

4 Are you currently targeting a daily calorie goal?
- ◯ Yes
- ◯ No

If yes, how many calories? __________________________________
(calories/day)

5 How often do you weigh yourself?
- ◯ Never
- ◯ Monthly
- ◯ Weekly
- ◯ Daily

6 Over the next 3 months, how interested are you in losing weight?

Not interested — Very Interested

(Place a mark on the scale above)

7 Over the next 3 months, how confident are you that you can lose weight?

Not confident — Very confident

(Place a mark on the scale above)

For each item, select the response that describes your motivation for weight loss.

I want to lose weight...

	Absolutely not	Somewhat	Moderately	Strongly
For health reasons	◯	◯	◯	◯
To decrease my health risks	◯	◯	◯	◯
To be eligible for total joint replacement surgery	◯	◯	◯	◯
To not attract attention	◯	◯	◯	◯
To be more appreciated/liked	◯	◯	◯	◯
To be more attractive	◯	◯	◯	◯
To be able to dress more fashionably	◯	◯	◯	◯
To feel more self-confident	◯	◯	◯	◯

9 How satisfied are you with your current weight?
- ◯ Very dissatisfied with my weight
- ◯ Somewhat dissatisfied with my weight
- ◯ Neither dissatisfied nor satisfied with my weight
- ◯ Somewhat satisfied with my weight
- ◯ Very satisfied with my weight

10 Please read through each description given below, pick the ONE description that best describes your regular daily activity and select that box (Check only one box).

◯ I am confined to bed all day.
◯ I am confined to bed most of the day except for minimal transfer activities (going to the bathroom, etc) ◯ I am either in bed or sitting in a chair most of the day.
◯ I sit most of the day, except for minimal transfer activities, no walking or standing.
◯ I sit most of the day, but I stand occasionally and walk a minimal amount in my house. (I may rarely leave the house for an appointment and may require the use of a wheelchair or scooter for transportation.)
◯ I walk around my house to a moderate degree but I don't leave the house on a regular basis. I may leave the house occasionally for an appointment.
◯ I walk around my house and go outside at will, walking one or two blocks at a time.
◯ I walk around my house, go outside at will and walk several blocks at a time without any assistance (weather permitting).
◯ I am up and about at will in my house and can go out and walk as much as I would like with no restrictions (weather permitting).
◯ I am up and about at will in my house and outside. I also work outside the house in a minimally active job ◯ I am up and about at will in my house and outside. I also work outside the house in a moderately active job ◯ I am up and about at will in my house and outside. I also work outside the house in an extremely active job
◯ I am up and about at will in my house and outside. I also participate in relaxed physical activity such as jogging, dancing, cycling, swimming occasionally (2-3 times per month) ◯ I am up and about at will in my house and outside. I also participate in relaxed physical activity such as jogging, dancing, cycling, swimming 2-3 times per week ◯ I am up and about at will in my house and outside. I also participate in relaxed physical activity such as jogging, dancing, cycling, swimming daily
◯ I am up and about at will in my house and outside. I also participate in vigorous physical activity such as competitive level sports occasionally (2-3 times per month)
◯ I am up and about at will in my house and outside. I also participate in vigorous physical activity such as competitive level sports occasionally 2-3 times per week
◯ I am up and about at will in my house and outside. I also participate in vigorous physical activity such as competitive level sports daily

Which joint are you interested in having replaced (with total joint arthroplasty)?

◯ Knee
◯ Hip

INSTRUCTIONS: This survey asks for your view about your knee. This information will help us keep track of how you feel about your knee and how well you are able to perform your usual activities.
Answer every question by ticking the appropriate box, only one box for each question. If you are unsure about how to answer a question, please give the best answer you can.

Symptoms
These questions should be answered thinking of your knee symptoms during the last week.

S1. Do you have swelling in your knee?
- ◯ Never
- ◯ Rarely
- ◯ Sometimes
- ◯ Often
- ◯ Always

S2. Do you feel grinding, hear clicking or any other type of noise when your knee moves?
- ◯ Never
- ◯ Rarely
- ◯ Sometimes
- ◯ Often
- ◯ Always

S3. Does your knee catch or hang up when moving?
- ◯ Never
- ◯ Rarely
- ◯ Sometimes
- ◯ Often
- ◯ Always

S4. Can you straighten your knee fully?
- ◯ Always
- ◯ Often
- ◯ Sometimes
- ◯ Rarely
- ◯ Never

S5. Can you bend your knee fully?
- ◯ Always
- ◯ Often
- ◯ Sometimes
- ◯ Rarely
- ◯ Never

Stiffness
The following questions concern the amount of joint stiffness you have experienced during the last week in your knee. Stiffness is a sensation of restriction or slowness in the ease with which you move your knee joint.

S6. How severe is your knee joint stiffness after first wakening in the morning?
- ◯ None
- ◯ Mild
- ◯ Moderate
- ◯ Severe
- ◯ Extreme

S7. How severe is your knee stiffness after sitting, lying or resting later in the day?
- ◯ None
- ◯ Mild
- ◯ Moderate
- ◯ Severe
- ◯ Extreme

Pain

P1. How often do you experience knee pain?
- ◯ Never
- ◯ Monthly
- ◯ Weekly
- ◯ Daily
- ◯ Always

What amount of knee pain have you experienced the last week during the following activities?

P2. Twisting/pivoting on your knee
- ◯ None
- ◯ Mild
- ◯ Moderate
- ◯ Severe
- ◯ Extreme

P3. Straightening knee fully
- ◯ None
- ◯ Mild
- ◯ Moderate
- ◯ Severe
- ◯ Extreme

P4. Bending knee fully
- ◯ None
- ◯ Mild
- ◯ Moderate
- ◯ Severe
- ◯ Extreme

P5. Walking on flat surface
- ◯ None
- ◯ Mild
- ◯ Moderate
- ◯ Severe
- ◯ Extreme

P6. Going up or down stairs
- ◯ None
- ◯ Mild
- ◯ Moderate
- ◯ Severe
- ◯ Extreme

P7. At night while in bed
- ◯ None
- ◯ Mild
- ◯ Moderate
- ◯ Severe
- ◯ Extreme

P8. Sitting or lying
- ◯ None
- ◯ Mild
- ◯ Moderate
- ◯ Severe
- ◯ Extreme

P9. Standing upright
- ◯ None
- ◯ Mild
- ◯ Moderate
- ◯ Severe
- ◯ Extreme

Function, daily living

The following questions concern your physical function. By this we mean your ability to move around and to look after yourself. For each of the following activities please indicate the degree of difficulty you have experienced in the last week due to your knee.

A1. Descending stairs
- ◯ None
- ◯ Mild
- ◯ Moderate
- ◯ Severe
- ◯ Extreme

A2. Ascending stairs
- ◯ None
- ◯ Mild
- ◯ Moderate
- ◯ Severe
- ◯ Extreme

For each of the following activities please indicate the degree of difficulty you have experienced in the last week due to your knee.

A3. Rising from sitting
- ◯ None
- ◯ Mild
- ◯ Moderate
- ◯ Severe
- ◯ Extreme

A4. Standing
- ◯ None
- ◯ Mild
- ◯ Moderate
- ◯ Severe
- ◯ Extreme

A5. Bending to floor/pick up an object
- ◯ None
- ◯ Mild
- ◯ Moderate
- ◯ Severe
- ◯ Extreme

A6. Walking on flat surface
- ◯ None
- ◯ Mild
- ◯ Moderate
- ◯ Severe
- ◯ Extreme

A7. Getting in/out of car
- ◯ None
- ◯ Mild
- ◯ Moderate
- ◯ Severe
- ◯ Extreme

A8. Going shopping
- ◯ None
- ◯ Mild
- ◯ Moderate
- ◯ Severe
- ◯ Extreme

A9. Putting on socks/stockings	◯ None<br>◯ Mild<br>◯ Moderate<br>◯ Severe<br>◯ Extreme
A10. Rising from bed	◯ None<br>◯ Mild<br>◯ Moderate<br>◯ Severe<br>◯ Extreme
A11. Taking off socks/stockings	◯ None<br>◯ Mild<br>◯ Moderate<br>◯ Severe<br>◯ Extreme
A12. Lying in bed (turning over, maintaining knee position)	◯ None<br>◯ Mild<br>◯ Moderate<br>◯ Severe<br>◯ Extreme
A13. Getting in/out of bath	◯ None<br>◯ Mild<br>◯ Moderate<br>◯ Severe<br>◯ Extreme
A14. Sitting	◯ None<br>◯ Mild<br>◯ Moderate<br>◯ Severe<br>◯ Extreme
A15. Getting on/off toilet	◯ None<br>◯ Mild<br>◯ Moderate<br>◯ Severe<br>◯ Extreme

For each of the following activities please indicate the degree of difficulty you have experienced in the last week due to your knee.

A16. Heavy domestic duties (movign heavy boxes, scrubbing floors, etc)	◯ None<br>◯ Mild<br>◯ Moderate<br>◯ Severe<br>◯ Extreme
A17. Light domestic duties (cooking, dusting, etc)	◯ None<br>◯ Mild<br>◯ Moderate<br>◯ Severe<br>◯ Extreme

Function, sports and recreational activities
The following questions concern your physical function when being active on a higher level. The questions should be answered thinking of what degree of difficulty you have experienced during the last week due to your knee.

SP1. Squatting
- ◯ None
- ◯ Mild
- ◯ Moderate
- ◯ Severe
- ◯ Extreme

SP2. Running
- ◯ None
- ◯ Mild
- ◯ Moderate
- ◯ Severe
- ◯ Extreme

SP3. Jumping
- ◯ None
- ◯ Mild
- ◯ Moderate
- ◯ Severe
- ◯ Extreme

SP4. Twisting/pivoting on your injured knee
- ◯ None
- ◯ Mild
- ◯ Moderate
- ◯ Severe
- ◯ Extreme

SP5. Kneeling
- ◯ None
- ◯ Mild
- ◯ Moderate
- ◯ Severe
- ◯ Extreme

Quality of Life

Q1. How often are you aware of your knee problem?
- ◯ Never
- ◯ Monthly
- ◯ Weekly
- ◯ Daily
- ◯ Constantly

Q2. Have you modified your life style to avoid potentially damaging activities to your knee?
- ◯ Not at all
- ◯ Mildly
- ◯ Moderately
- ◯ Severely
- ◯ Totally

Q3. How much are you troubled with lack of confidence in your knee?
- ◯ Not at all
- ◯ Mildly
- ◯ Moderately
- ◯ Severely
- ◯ Totally

Q4. In general, how much difficulty do you have with your knee?

◯ None
◯ Mild
◯ Moderate
◯ Severe
◯ Extreme

INSTRUCTIONS: This section asks for your view about your hip. This information will help us keep track of how you feel about your hip and how well you are able to do your usual activities.
Answer every question by ticking the appropriate circle, only one circle for each question. If you are uncertain about how to answer a question, please give the best answer you can.

Symptoms

These questions should be answered thinking of your hip symptoms and difficulties during the last week.

S1. Do you feel grinding, hear clicking or any other type of noise from your hip?

◯ Never ◯ Rarely ◯ Sometimes ◯ Often ◯ Always

S2. Difficulties spreading legs wide apart

◯ None ◯ Mild ◯ Moderate ◯ Severe ◯ Extreme

S3. Difficulties to stride out when walking

◯ None ◯ Mild ◯ Moderate ◯ Severe ◯ Extreme

Stiffness

The following questions concern the amount of joint stiffness you have experienced during the last week in your hip. Stiffness is a sensation of restriction or slowness in the ease with which you move your hip joint.

S4. How severe is your hip joint stiffness after first wakening in the morning?

◯ None ◯ Mild ◯ Moderate ◯ Severe ◯ Extreme

S5. How severe is your hip stiffness after sitting, lying or resting later in the day?

◯ None ◯ Mild ◯ Moderate ◯ Severe ◯ Extreme

Pain

P1. How often is your hip painful?

◯ Never ◯ Monthly ◯ Weekly ◯ Daily ◯ Always

What amount of hip pain have you experienced the last week during the following activities?

P2. Straightening your hip fully

◯ None ◯ Mild ◯ Moderate ◯ Severe ◯ Extreme

P3. Bending your hip fully

◯ None ◯ Mild ◯ Moderate ◯ Severe ◯ Extreme

P4. Walking on a flat surface

◯ None ◯ Mild ◯ Moderate ◯ Severe ◯ Extreme

P5. Going up or down stairs

◯ None ◯ Mild ◯ Moderate ◯ Severe ◯ Extreme

P6. At night while in bed

◯ None ◯ Mild ◯ Moderate ◯ Severe ◯ Extreme

P7. Sitting or lying

◯ None ◯ Mild ◯ Moderate ◯ Severe ◯ Extreme

P8. Standing upright

◯ None ◯ Mild ◯ Moderate ◯ Severe ◯ Extreme

P9. Walking on a hard surface (asphalt, concrete, etc.)

◯ None ◯ Mild ◯ Moderate ◯ Severe ◯ Extreme

P10. Walking on an uneven surface

◯ None ◯ Mild ◯ Moderate ◯ Severe ◯ Extreme

Function, daily living

The following questions concern your physical function. By this we mean your ability to move around and to look after yourself. For each of the following activities please indicate the degree of difficulty you have experienced in the last week due to your hip.

A1. Descending stairs

◯ None ◯ Mild ◯ Moderate ◯ Severe ◯ Extreme

A2. Ascending stairs

◯ None ◯ Mild ◯ Moderate ◯ Severe ◯ Extreme

A3. Rising from sitting

◯ None ◯ Mild ◯ Moderate ◯ Severe ◯ Extreme

A4. Standing

◯ None ◯ Mild ◯ Moderate ◯ Severe ◯ Extreme

A5. Bending to the floor/pick up an object

◯ None ◯ Mild ◯ Moderate ◯ Severe ◯ Extreme

A6. Walking on a flat surface

◯ None ◯ Mild ◯ Moderate ◯ Severe ◯ Extreme

A7. Getting in/out of car

◯ None ◯ Mild ◯ Moderate ◯ Severe ◯ Extreme

A8. Going shopping

◯ None ◯ Mild ◯ Moderate ◯ Severe ◯ Extreme

A9. Putting on socks/stockings

◯ None ◯ Mild ◯ Moderate ◯ Severe ◯ Extreme

A10. Rising from bed

◯ None ◯ Mild ◯ Moderate ◯ Severe ◯ Extreme

A11. Taking off socks/stockings

◯ None ◯ Mild ◯ Moderate ◯ Severe ◯ Extreme

A12. Lying in bed (turning over, maintaining hip position)

◯ None ◯ Mild ◯ Moderate ◯ Severe ◯ Extreme

A13. Getting in/out of bath

◯ None ◯ Mild ◯ Moderate ◯ Severe ◯ Extreme

A14. Sitting

◯ None ◯ Mild ◯ Moderate ◯ Severe ◯ Extreme

A15. Getting on/off toilet

◯ None ◯ Mild ◯ Moderate ◯ Severe ◯ Extreme

A16. Heavy domestic duties (moving heavy boxes, scrubbing floors, etc.)

◯ None ◯ Mild ◯ Moderate ◯ Severe ◯ Extreme

A17. Light domestic duties (cooking, dusting, etc.)

◯ None ◯ Mild ◯ Moderate ◯ Severe ◯ Extreme

Function, sports and recreational activities

The following questions concern your physical function when being active on a higher level. The questions should be answered thinking of what degree of difficulty you have experienced during the last week due to your hip.

SP1. Squatting

◯ None ◯ Mild ◯ Moderate ◯ Severe ◯ Extreme

SP2. Running

◯ None ◯ Mild ◯ Moderate ◯ Severe ◯ Extreme

SP3. Twisting/pivoting on loaded leg

◯ None ◯ Mild ◯ Moderate ◯ Severe ◯ Extreme

SP4. Walking on uneven surface

◯ None ◯ Mild ◯ Moderate ◯ Severe ◯ Extreme

Quality of Life

Q1. How often are you aware of your hip problem?

◯ Never ◯ Monthly ◯ Weekly ◯ Daily ◯ Constantly

Q2. Have you modified your life style to avoid activities potentially damaging to your hip?

◯ Not at all ◯ Mildly ◯ Moderately ◯ Severely ◯ Totally

Q3. How much are you troubled with lack of confidence in your hip?

◯ Not at all ◯ Mildly ◯ Moderately ◯ Severely ◯ Extremely

Q4. In general, how much difficulty do you have with your hip?

◯ None ◯ Mild ◯ Moderate ◯ Severe ◯ Extreme

Overall Health

In general, would you say your health is:

◯ Excellent
◯ Very good
◯ Good
◯ Fair
◯ Poor

In general, would you say your quality of life is:

◯ Excellent
◯ Very good
◯ Good
◯ Fair
◯ Poor

In general, how would you rate your physical health?	◯ Excellent<br>◯ Very good<br>◯ Good<br>◯ Fair<br>◯ Poor
In general, how would you rate your mental health, including your mood and your ability to think?	◯ Excellent<br>◯ Very good<br>◯ Good<br>◯ Fair<br>◯ Poor
In general, how would you rate your satisfaction with your social activities and relationships?	◯ Excellent<br>◯ Very good<br>◯ Good<br>◯ Fair<br>◯ Poor
In general, please rate how well you carry out your usual social activities and roles. (This includes activities at home, at work and in your community, and responsibilities as a parent, child, spouse, employee, friend, etc.)	◯ Excellent<br>◯ Very good<br>◯ Good<br>◯ Fair<br>◯ Poor
To what extent are you able to carry out your everyday physical activities such as walking, climbing stairs, carrying groceries, or moving a chair?	◯ Completely<br>◯ Mostly<br>◯ Moderately<br>◯ A little<br>◯ Not at all
In the past 7 days<br>How often have you been bothered by emotional problems such as feeling anxious, depressed or irritable?	◯ Never<br>◯ Rarely<br>◯ Sometimes<br>◯ Often<br>◯ Always
In the past 7 days<br>How would you rate your fatigue on average?	◯ None<br>◯ Mild<br>◯ Moderate<br>◯ Severe<br>◯ Very severe
In the past 7 days<br>How would you rate your pain on average?	◯ 0 (No pain)<br>◯ 1<br>◯ 2<br>◯ 3<br>◯ 4<br>◯ 5<br>◯ 6<br>◯ 7<br>◯ 8<br>◯ 9<br>◯ 10 (Worst Imaginable Pain)

How often do you do or experience the following?

	Never do this	Rarely do this	Sometimes do this	Often do this	Regularly do this as part of my routine
I enjoy myself when I exercise.	○	○	○	○	○
I perform strength training exercises twice a week.	○	○	○	○	○
I am optimistic about the day.	○	○	○	○	○
I like to try new activities.	○	○	○	○	○
I have a friend who I know energizes me.	○	○	○	○	○
I have identified at least one activity that brings me joy and energy.	○	○	○	○	○
I am involved with a group (activity, exercise class, art class, religious affiliation or the like)	○	○	○	○	○

How often do you do or experience the following?

	Never do this	Rarely do this	Sometimes do this	Often do this	Regularly do this as part of my routine
I eat 4 fruits a day.	○	○	○	○	○
I eat 5 or more vegetables a day.	○	○	○	○	○
I know proper portions for protein, carbohydrates, and fats, and I eat those portions.	○	○	○	○	○
I think about the food that I eat and ask myself if it is good for my body.	○	○	○	○	○
I view food as fuel, as medicine, and enjoyment too.	○	○	○	○	○

How often do you do or experience the following?

	Never do this	Rarely do this	Sometimes do this	Often do this	Regularly do this as part of my routine
I set long-term goals for myself, share them with someone, and review them.	○	○	○	○	○
I set monthly goals and share them with someone.	○	○	○	○	○
I set weekly goals and share them with someone.	○	○	○	○	○
I set daily goals for myself and keep myself accountable for them.	○	○	○	○	○

14. During the past month, how often did you drink each of the following beverages. A serving is one 8-ounce glass or can of the drink or beverage. If you drink a 16-ounce bottle, please count that as 2 servings.

	Less than once per week	Once per week	2 to 4 times per week	Nearly daily or daily	Twice or more per day
100% fruit juice (e.g. apple, grape, orange)	○	○	○	○	○
Soda with sugar (e.g. Coke, Pepsi, Sprite)	○	○	○	○	○
Other drink with sugar (e.g. sweetened iced tea, gatorade, fruit punch, fruit cocktail)	○	○	○	○	○
Diet soda (e.g. Diet Coke, Diet Pepsi, Diet Sprite)	○	○	○	○	○
Other flavored drink without sugar (e.g. sugar-free iced tea, Crystal Light)	○	○	○	○	○

15 During the past month, on average, how many times did you eat breakfast, lunch, or dinner from fast food restaurants such as McDonald's, Burger King, Wendy's, Arby's, Pizza Hut, or Kentucky Fried Chicken?

○ Never ○ 1 to 3 times in the past month
○ 1 or 2 times per week
○ 3 or 4 times per week
○ 5 or 6 times per week
○ 7 or more times per week

During the past month, how often did you eat each of the following foods?

	Less than once per week	Once per week	2 to 4 times per week	Nearly daily or daily	Twice or more per day
Whole milk dairy foods (whole milk, hard cheese, butter, ice cream)	◯	◯	◯	◯	◯
Low-fat milk products (for example, low-fat/skim milk, yogurt, cottage cheese)	◯	◯	◯	◯	◯
Whole grain foods (e.g. whole grain breads, brown rice)	◯	◯	◯	◯	◯
Pasta, rice, noodles	◯	◯	◯	◯	◯
Baked products (donuts, cookies, muffins, crackers, cakes, sweet rolls, pastries)	◯	◯	◯	◯	◯
Deep fried foods (deep fried chicken, fish or seafood, french fries, onion rings)	◯	◯	◯	◯	◯
Vegetables (fresh, frozen, or canned)	◯	◯	◯	◯	◯
Fruit (fresh, frozen, or canned)	◯	◯	◯	◯	◯
Fish (not fried)	◯	◯	◯	◯	◯

15 Do you currently smoke tobacco on a daily basis, less than daily, or not at all?

◯ Daily ◯ Less than daily
◯ Not at all

18 How often do you have a drink containing alcohol?

◯ Never ◯ Monthly or less
◯ 2 to 4 times a month
◯ 2 to 3 times a week ◯ 4 or more times a week

18 How many drinks containing alcohol do you have on a typical day when you are drinking?

◯ 1 or 2 ◯ 3 or 4 ◯ 5 or 6
◯ 8, or 9 ◯ 10 or more

20 During the past month, on average, how many hours per day did you spend watching TV or DVDs/Videos?

____
(hours per day)

20 During the past month, on average, how many hours per day did you spend using the computer? (Do not include time spent at work)

____
(hours per day)

21 During the past month, on average, how many hours per week did you spend engaged in walking for leisure?

____
(hours per week)

21 During the past month, on average, how many hours per week did you spend engaged in light or moderate recreational activities or sports such as bowling, yoga, stretching classes, skating, or other similar activities? (Do not include walking.)

____
(hours per week)

21 During the past month, on average, how many hours per week did you spend engaged in vigorous recreational activities or sports such as jogging, swimming, cycling, aerobic dance, skiing, or other similar activities?

____
(hours per week)

21 During the past month, on average, how many hours per week did you spend engaged in resistance training or weight lifting?

____
(hours per week)

22 Do you use any tracking device to measure your level of physical activity?

○ Yes ○ No

23 During the past month, how many hours of sleep do you get in an average 24 hour period?

____

24 What is your age?

____
(years)

25 What is your gender?

○ Female ○ Male ○ Additional gender category ○ Prefer not to answer

Additional gender category:

____

26 Which of the following best describes your race? (Please check all that apply.)

☐ White or Caucasian ☐ Hispanic or Latino ☐ Black or African American ☐ Asian ☐ American Indian or Alaskan Native ☐ Native Hawaiian or Pacific Islander ☐ Other

Other race:

____

27 What is the highest level of education that you have completed? (Please check only one.)

○ Some high school ○ High school graduate ○ Some college ○ College graduate ○ Graduate school

28 What is your marital status? (Please check only one.)

○ Single ○ living with a partner or significant other ○ Married ○ Separated/Divorced ○ Widowed

29	During the past year, what was the total income of your household before taxes? Please include money from all sources such as salaries, tips, Social Security, Transitional AFDC (TAFDC), retirement, and any other kind of support.	◯ $5,000 or Less ◯ $5,001 to $10,000 ◯ $10,001 to $20,000 ◯ $20,001 to $40,000 ◯ $40,001 to $70,000 ◯ $70,001 to $100,000 ◯ $100,001 to $150,000 ◯ More than $150,000 ◯ Don't Know
30	What is your employment status?	◯ Student ◯ Working full time ◯ Working part time ◯ Not working, but looking for work ◯ Not working, not looking for work
